# Symptom Persistence and Memory Performance in Posttraumatic Stress Disorder: A Gene X Environment Pilot Stud

**DOI:** 10.3390/bs2020103

**Published:** 2012-06-01

**Authors:** Annie-Claude David, Geeta A. Thakur, Vivian Akerib, Jorge Armony, Isabelle Rouleau, Alain Brunet

**Affiliations:** 1 Department of Psychology, Université du Québec à Montréal, Montreal H2X 1L7, Canada; E-Mails: annieclaude.david@gmail.com (A.-C.D.); rouleau.isabelle@uqam.ca (I.R.); 2 Douglas Mental Health University Institute, Montreal H4H 1R2, Canada; E-Mails: geeta.thakur@douglas.mcgill.ca (G.A.T.); vakerib@videotron.ca (V.A.); jorge.armony@mcgill.ca (J.A.); 3 Department of Psychiatry, McGill University, Montreal H3A 0G4, Canada

**Keywords:** FKBP5, HPA-axis, glucocorticoid receptor, remission, Canadians

## Abstract

The FKBP5 gene, a glucocorticoid receptor (GR)-regulating co-chaperone of stress proteins, is of special interest because of its role in hypothalamic-pituitary-adrenal (HPA)-axis regulation. However, studies finding a genetic relationship between posttraumatic stress disorder (PTSD) and the FKBP5 gene have failed to distinguish between the development and persistence of PTSD, thereby limiting the prognostic usefulness of such a finding. The present study sought to longitudinally explore this question by examining the association between four single-nucleotide polymorphisms (SNPs) in the FKBP5 gene (rs3800373, rs9470080, rs1360780, and rs9296158), the persistence of PTSD (severity and diagnostic status), and memory performance among twenty-two treatment-seekers diagnosed with acute PTSD. Results showed that the four SNPs significantly interacted with improvement in PTSD symptoms as well as PTSD diagnostic status. Individuals homozygous for the dominant allele and having experienced higher levels of peritraumatic responses subsequently showed more memory dysfunction. The results of this study suggest that SNPs in the FKBP5 gene are associated with symptom persistence and memory dysfunction in acute PTSD.

## 1. Introduction

There are two important questions when trying to understand Posttraumatic Stress Disorder (PTSD) [1]. The first one is why do some trauma-exposed individuals *develop* the disorder while others do not? And the second one is why do some *remit* from the disorder while others do not? The latter echoes the observation that remission from PTSD in the months following exposure to traumatic stress is the norm [2], and that PTSD is considered a disorder of failed recovery [3].

In a related vein, from a conceptual point of view, a good predictor of the development of PTSD should be a poor predictor of its persistence, as many PTSD cases remit within the first year or so [4,5]. If this account is correct, we would expect to find different sets of gene x environment (G × E) interactions as a function of time elapsed since the development of the disorder [6]. One set of G × E might explain the development of the disorder (*i.e*., answers the first question), while another set might explain its persistence (*i.e*., answers the second question). It also follows that genetic studies with a cross-sectional design cannot uncover such interactions [6] and that it is probably more important from a clinical perspective to uncover G × E interactions related to the persistence of the disorder. Distinguishing between the persistence and remission of PTSD, which previous genetic studies have failed to do, calls for a longitudinal investigation.

As PTSD is the product of complex interplay of genetic and environmental factors, the study of endophenotypes may represent a more proximal indicator of genotype [6]. Endophenotypes are characterized as neurophysiological, biochemical, endocrinological, neuroanatomical, cognitive, or neuropsychological constructs mediating the relationship between genotype and expressed syndromes [7]. A literature search in popular databases such as PubMed or P.I.L.O.T.S. suggests that the role of G × E interactions in the development of PTSD—and even more so in its persistence—has been largely neglected thus far.

### 1.1. Glucocorticoids and PTSD

One important endophenotype mediating the long-term effects of negative life events on the development of psychiatric disorders is the hormonal system. Stress hormones, such as glucocorticoids produced by the stress-responsive hypothalamic-pituitary-adrenal (HPA) axis, are well recognized for their regulatory role in peripheral metabolism and various brain functions. Glucocorticoids regulate the physiologic stress response by preparing the end organs for a fight-or-flight response, but they are also critical for terminating the response via a negative feedback loop of the HPA-axis through the activation of the glucocorticoid receptors [8]. Prolonged or excessive activation of this system has been implicated in the pathogenesis of various mood and anxiety disorders, including PTSD [8]. The FKBP5 gene, a glucocorticoid receptor (GR)-regulating co-chaperone of stress proteins, is of special interest because of its role in HPA-axis regulation [8].

### 1.2. Glucocorticoids and Traumatic Memory

Another important endophenotype associated with PTSD is memory disturbances [9]. PTSD is characterized by both involuntary recollection of the trauma in the form of intrusive thoughts, nightmares, and vivid sensory memories (“flashbacks”) as well as memory deficits. It has been proposed that high or low levels of cortisol existing over a long period of time can injure the hippocampus and contribute to memory disturbances [10]. Finally, research has implicated glucocorticoids in the modulation of extinction of fear memories [11]. Thus, converging evidence suggests that glucocorticoids are involved in memory dysfunction, which could interfere with extinction learning, and therefore explain, in part, the persistence of the disorder. If this is true, then individual differences in HPA-axis functioning may interact with trauma exposure and memory to confer higher risk for the persistence of PTSD.

### 1.3. FKBP5 Gene and PTSD

Only a few studies have investigated the association between single-nucleotide polymorphisms (SNPs) in the FKBP5 gene and PTSD. Koenen *et al*. [12] found that the *C* allele of the rs3800373 and the *T* allele of the rs1360780, two potentially functional SNPs, were associated with increased peritraumatic dissociation in 46 children after medical trauma [12]. Unfortunately, no measure of PTSD symptom or diagnosis was used in this study. A cross-sectional study reported that four FKBP5 SNPs (rs9296158, rs3800373, rs1360780, and rs9470080) interacted with childhood abuse to modify the severity of adult PTSD symptoms in a population of 900 urban, low-income, predominantly black men and women [13]. The *A* allele of rs9296158, the *C* allele of rs3800373, the *T* allele of rs1360780, and the *T* allele of rs9470080 were associated with a higher risk for current PTSD symptoms in the presence of severe child abuse. However, it is unclear if this finding applies to the development or persistence of PTSD due to lack of examination of the time elapsed since the index trauma. Further, not all subjects met the diagnostic criteria for current PTSD obscuring the implications of this finding. Using a subset of the same sample, Mehta *et al*. found that allele A carriers of rs9296158 showed GR supersensitivity with PTSD while baseline cortisol levels were decreased in PTSD only in patients with GG genotype [14]. In another recent study, Xie *et al*. investigated the interactive effects of FKBP5 SNPs and childhood adversity on the risk for lifetime PTSD in two populations: African Americans and European Americans totalling 2427 participants [15]. African Americans carrying the TT genotype of the rs9470080 SNP had the lowest risk for PTSD if they had never experienced any childhood adversity. However, they had the highest risk for lifetime PTSD after exposure to childhood adversity. These results provide further evidence for a GxE effect of FKBP5 and childhood abuse on the risk for lifetime PTSD, but fails to address the question as to whether FKBP5 is associated with the development or the persistence of PTSD. Furthermore, because many of the participants were cocaine, opioid, or alcohol dependence patients, the external validity of such results is limited and awaits further replication. Finally, Sarapas *et al*. found that homozygosity of any recessive allele (rs9296158, rs3800373, rs1360780, and rs9470080) predicted lower expression of FKBP5, which was in turn associated with both lower cortisol and higher PTSD symptom severity [16].

In sum, the available cross-sectional evidence suggests the presence of a small interactive effect of FKBP5 polymorphisms and childhood adversity on the risk for PTSD symptoms or disorder in very large samples of adults. No study however has adressed whether the gene was associated with the development or the persistence of the disorder, and this probably explains why very large samples were needed to obtain a significant result. According to the literature, the FKBP5 gene is associated with PTSD, perhaps via memory disturbances. If that is the case, particular genotypes in individuals suffering from acute PTSD would confer risk of chronic unremitting PTSD.

### 1.4. Goal and Hypotheses

A group of treatment-seekers, first time sufferers from acute PTSD, at risk for developing a more chronic form of PTSD, were followed prospectively over 6 months. The aim was to investigate if certain SNPs in the FKBP5 gene were involved in the persistence of the disorder. We hypothesized that (i) there would be an association between PTSD diagnostic status measured 9 months after trauma-exposure (*i.e*., 6 months after enrollment in the study) and the FKBP5 gene, thereby suggesting a G × E interaction conferring greater risk for chronicity of PTSD, all other variables being equal; that (ii) the FKBP5 gene would prospectively explain improvement in PTSD symptoms; and that (iii) subjective severity of trauma as measured by peritraumatic distress and dissociation and genetic influences (FKBP5) would have an interactive effect on endophenotypes such as memory dysfunction in chronic unremitting PTSD.

## 2. Methods

### 2.1. Participants

Thirty-nine treatment-seeking individuals meeting the DSM-IV-TR [17] criteria for acute PTSD (*i.e*., PTSD of less than 3 months duration) were recruited at a private specialized clinic in the context of a longitudinal study investigating neuropsychological functioning and the remission processes of PTSD. Among these, 24 provided a saliva sample for DNA extraction (an optional part of the study). Twenty-two of them, aged 21–48 years old (*M* = 33, *SD* = 9.8), mostly Caucasians, were available for follow-up 6 months later. The study completers included 13 individuals who remitted from their PTSD during the study and another 9 who did not. The traumatic events were comprised of physical or sexual assault (*n* = 11), motor vehicle accidents (*n* = 6), fire or explosion (*n* = 3), witnessing a violent death (*n* = 1), and a work-related accident (*n* = 1). Demographic and clinical variables are presented in Table 1.

#### 2.1.1. Inclusion and Exclusion Criteria

The inclusion criteria consisted of: (i) being 18–65 years old; (ii) satisfying the DSM-IV-TR [17] criteria for acute PTSD; and (iii) living less than an hour drive away from the study recruitment site (for convenience purposes). Participants were excluded if they (i) understood neither French nor English; (ii) had or were suspected of having a history of traumatic brain injury; (iii) had a lifetime diagnosis of psychosis, substance or alcohol dependence, bipolar disorder, a lifetime diagnosis of PTSD prior to their current diagnosis of acute PTSD; (iv) had been clinically depressed in the last 2 years; or (v) were taking psychotropic medication at the time of recruitment.

**Table 1:** Clinical data for the remitted( *n* = 13) and persistent (*n* = 9) PTSD groups.

Measure	Trauma + 3 months		Trauma + 9 months	
	*M*	*SD*	*M*	*SD*
Depression score^1 ^:				
Remitted PTSD group	9.85	4.93	4.92	5.30
Persistent PTSD group	12.67	8.97	9.89	5.93
	*n*(%)		*n*(%)	
Participants with a comorbid disorder:				
Remitted PTSD group	9 (41)		2 (9)	
Persistent PTSD group	8 (36)		7 (32)*	
Participants taking psychotropic medication:				
Remitted PTSD group	4 (18)		3 (14)	
Persistent PTSD	5 (23)		5 (23)	
Anti-depressants	5 (23)		7 (32)	
Benzodiazepines	2 (9)		0	
Opioid analgesics	1 (4.5)		0	
Anti-psychotics	1 (4.5)		1 (4.5)	

^1^—Beck depression inventory; * *p* < 0.05.

### 2.2. Materials and Apparatus

#### 2.2.1. Trauma-Related and Other Clinical Measures

The Peritraumatic Distress Inventory (PDI; [18,19] is a 13-item self-report measure assessing the degree of distress experienced during and immediately after a traumatic event. Items are rated on a 5-point Likert scale ranging from 0 (not at all true) to 4 (extremely true). The total score is obtained by summing the responses across all items. The Peritraumatic Dissociation Experience Questionnaire (PDEQ; [20,21] is a self-report questionnaire assessing the degree of dissociation experienced during and immediately after the trauma. Items are rated on a 5-point Likert scale ranging from 1 (not at all true) to 5 (extremely true). The sum of all 10 items provides a total score. The Clinician-Administered PTSD Scale (CAPS; [22] is a semi-structured diagnostic interview assessing the frequency and intensity of the 17 symptoms of DSM-IV-TR [15] PTSD during the last month. The CAPS was used by a trained master-level clinician to confirm the PTSD diagnosis. Presence of symptoms (*i.e*., frequency and intensity) was ascertained according to the conventional 1–2 CAPS rule [23]. A diagnosis of PTSD was conferred using the DSM-IV-TR rules [17], and if the CAPS score (the sum of all items) was superior to 45. Remission from PTSD was defined as no longer meeting the DSM-IV-TR [17] diagostic criteria for PTSD and required a CAPS score inferior to 45 at nine months post-trauma. Psychiatric comorbidity and depression were assessed with the Mini International Neuropsychiatric Interview [24] and the Beck Depression Inventory (BDI; [25]. The MINI is a 30 min abbreviated psychiatric structured interview assessing the major adult axis I disorders of DSM-IV-TR [17], including substance and alcohol use.

#### 2.2.2. Memory-Related Measures

The Rey Auditory Verbal Learning Test (RAVLT) is a standardized measure of explicit verbal learning and memory performance [26]. It was used to assess immediate and delayed recall, recall with retroactive interference, recognition and cumulative learning [27]. The Aggie Figures Learning Test (AFLT; [28] was used to provide a nonverbal equivalent to the RAVLT. A memory composite score was computed from both instruments in order to have a more sensitive memory measure.

### 2.3. Procedure

Ethics approval was obtained from McGill University. Subject recruitment involved some (but not all) therapists from Traumatys Inc. asking their new patients if they would agree to receive a phone call from a research psychologist seeking their participation in a study. The number of participants approached is unknown. However, Traumatys treats about 200 patients per year across the province of Québec, and recruitment lasted 26 months. Participants gave written informed consent and received a small monetary compensation ($50) for each visit. Participants were tested at the onset of their therapy (henceforth referred to as Time 1 or T1) and 6 months later (henceforth called T2 or follow-up). Based on their PTSD diagnosis at T2, participants were assigned to one of two groups: (i) unremitting PTSD; or (ii) remitted PTSD. Each testing session required 3.5 h and was conducted in French or in English by a trained master-level clinical psychologist at the Douglas Mental Health University Institute.

### 2.4. DNA Extraction for Genetic Analyses

Saliva samples were frozen at −78 degrees celsius until assayed. DNA was extracted from saliva samples using Oragene kits. The FKBP5 polymorphisms were genotyped at Génome Québec using medium-scale SNP technologies (Sequenom iPlex Gold Technology). Distribution of the FKBP5 genotypes did not depart from Hardy-Weinberg equilibrium (rs3800373: *x*^2^ = 0.40, *p* = 0.53; rs9470080: *x*^2^ = 0.58, *p* = 0.45; rs1360780: *x*^2^ = 0.27, *p* = 0.70; rs9296158: *x*^2^ = 1.22, *p* = 0.27).

### 2.5. Data Analysis

The two study groups were first compared on demographic and clinical characteristics using *t*-tests or chi-square analyses, as appropriate. Then, the relationship between PTSD and FKBP5 polymorphisms was examined using a categorical approach (diagnostic status based on the CAPS) involving chi-square analyses. A series of linear regression analyses were subsequently performed to explore the contribution of FKBP5 polymorphisms on the improvement in PTSD symptoms according to the CAPS. Finally, using partial correlations, the interactive effect of environmental factors (subjective severity of trauma/peritraumatic responses) with genetic influences (FKBP5) on memory performance (endophenotype), which were previously shown to be associated with chronic unremitting PTSD [29], were investigated.

Immediate and delayed recall scores of both memory measures, which correlated significantly among each other, were transformed into *z*-scores in order to compute a memory composite score. The mean of the four *z*-scores was chosen rather than the sum because it allowed better handling of the few missing data points at T2 (*n* = 2). There were missing data for one of the SNPs (*n* = 1) due to DNA extraction difficulties making the results for that one SNP weaker. All tests were two-sided except for chi-square analyses because of their unilateral distribution, and the alpha level was set at 0.05. Considering the exploratory nature of this study, no correction for multiple testing was used.

## 3. Results

### 3.1. Clinical and Sociodemographic Data

Results of the interviewer-based CAPS (*t*[20] = 2.10, *p =* 0.05) almost reached significance with higher initial PTSD symptom score for chronic unremitted participants (*M* = 95, *SD* = 18) compared to individuals who remitted from the disorder at T2 (*M* = 79, *SD* = 17). Likewise, no between-group differences were found for peritraumatic dissociation (*t*[20] = 0.60, *p >* 0.05), peritraumatic distress (*t*[20] = 0.39, *p >* 0.05), gender (χ^2^[1] = 0.02, *p >* 0.05), ethnicity (χ^2^[1] = 1.5, *p >* 0.05), social status (χ^2^[1] = 0.20, *p >* 0.05), and level of education (χ^2^[1] = 0.28, *p >* 0.05). However, the remitted group was significantly younger (*M* = 29.6, *SD* = 10.1) than the non-remitting one (*M* = 38.8, *SD* = 6.5), *t*(20) = 2.39, *p =* 0.03. 

Many participants (77%) suffered from psychiatric comorbidity at T1 including major depressive episode (18%), hypomanic episode (4.5%), panic disorder (32%), agoraphobia (9%), social phobia (9%), obsessive compulsive disorder (9%), alcohol dependence (4.5%), drug dependence (4.5%), drug abuse (4.5%), bulimia (4.5%), and generalized anxiety disorder (4.5%). At T2, 41% still suffered from psychiatric comorbidity including major depressive episode (9%), panic disorder (14%), agoraphobia (18%), social phobia (4.5%), obsessive-compulsive disorder (9%), and generalized anxiety disorder (9%). Participants were all treatment seekers, thereby explaining the high level of comorbidity. At T1, groups did not differ on the number of comorbid disorders *t*(20) = 1.7, *p>* 0.05.The chronic group had more comorbid disorders at T2 compared to the remitted group *t*(20) = 3.3, *p* < 0.05. Types of medication and depressive symptoms are presented in Table 1.

### 3.2. Association Between PTSD Diagnostic Status and FKBP5 Polymorphisms

Because of the small sample size, recessive alleles were combined and genotype frequencies were compared using the following model: rs3800373: *AC* + *CCvs*. *AA*; rs9470080: *CT* + *TTvs*. *CC*; rs1360780: *CT* + *TTvs*. *CC*; rs9296158: *AG* + *AAvs*. *GG*. Analyses were then conducted according to PTSD diagnostic status at T2 (remitted *vs*. unremitting PTSD). Each *C* allele of rs3800373 (χ^2^[1] = 8.95, *p=* 0.001), each *T* allele of rs9470080 (χ^2^[1] = 6.77, *p=* 0.004), each *T* allele of rs1360780 (χ^2^[1] = 9.78, *p* < 0.001), and each *A* allele of rs9296158 (χ^2^[1] = 9.78, *p* < 0.001) was associated with a remitted PTSD diagnosis. These results suggest that the *AA* genotype at rs3800373, the *CC* genotype at rs9470080, the *CC* genotype at rs1360780 and the *GG* genotype at rs9296158 are associated with unremitting chronic PTSD.

### 3.3. Association Between FKBP5 Polymorphisms and Improvement in PTSD Symptoms

Our second hypothesis was that FKBP5 polymorphisms would predict improvement in PTSD symptoms. In order to test this, we created two new variables to be used in a stepwise multiple regression analysis. For each participant two ‘improvement’ scores were created by subtracting the latest PTSD symptom score from the earliest one on the interviewer-based CAPS PTSD symptom score (covering the last month). We then conducted a linear regression analysis with improvement in CAPS PTSD symptoms as the dependent variable. We simultaneously entered ‘classical’ predictors of chronic PTSD, *i.e*., the score of peritraumatic dissociation, initial CAPS PTSD symptom scores and gender as well as age because of the between-group difference observed with this variable. This first step explained 23% of the variance, *F*(4, 20) = 1.21, *p* = 0.35. There was, however, no significant predictor at this stage. As a second step, we added one FKBP5 polymorphism. In each of the four regression analyses, the only significant predictors were the FKBP5 polymorphisms. The betas of each FKBP5 SNP entered in the second step of their respective regression analysis to predict improvement in CAPS PTSD symptoms are presented in Table 2 along with the variance explained. Similar results were found when peritraumatic distress was used as a predictor instead of peritraumatic dissociation.

**Table 2:** Association of FKBP5 SNPs with improvement in PTSD symptom scores ^1^.

*SNP*	*B*	*SE B*	*ß*	*R^2^*
rs3800373	11.61	5.19	0.52*	0.43
rs9470080	8.05	5.1	0.38	0.34
rs1360780	11.38	4.96	0.53*	0.43
rs9296158	11.38	4.96	0.53*	0.43

^1^-Measured with the Clinician-administered PTSD Scale (CAPS); * *p* < 0.05.

### 3.4. Relation Between FKBP5 Gene, Peritraumatic Responses and Memory Performance

Next, we explored if memory performance was related to the FKBP5 gene. Because FKBP5 alone was not found to be associated with memory performance at T2, neither for rs3800373 (*r* = .03, *p* = 0.92), rs9470080 (*r* = 0.02, *p* = 0.94), rs1360780 (*r* = 0.05, *p* = 0.83), nor rs9296158 (*r* = 0.05, *p* = 0.83) and based on previous data supporting an interaction between FKBP5 and subjective severity of the trauma [13], we investigated the interactive effect of peritraumatic responses and genetic influences (FKBP5) on memory functioning. In order to do that, recessive homozygous and heterozygous genotypes of each polymorphism (rs3800373: *Ac*, *cc*; rs9470080: *Ct*, *tt*; rs1360780: *Ct*, *tt*; rs9296158: *Ga*, *aa*) were coded 1 and (dominant) homozygous genotypes of each polymorphism (rs3800373: *AA*; rs9470080: *CC*; rs1360780: *CC*; rs9296158: *GG)* were coded −1. A new “G × E interaction” variable was then created by computing the product of peritraumatic distress or dissociation with each polymorphism of the FKBP5 gene. We then explored the association between the new “G × E interaction” variable and memory performance at T2 with partial correlations controlling for main effects.

Significant partial correlations were found between the new “G × PDI” (*i.e*., peritraumatic distress) variable and memory performance for the 4 SNPs: rs3800373 *(r* = 0.52, *p* = 0.03), rs9470080 (*r* = 0.50, *p* = 0.04), rs1360780 (*r* = 0.51, *p* = 0.03), and rs9296158 (*r* = 0.51, *p* = 0.03). These results suggest an interactive effect of FKBP5 SNPs and subjective trauma severity on memory performance. We then conducted post-hoc analyses in order to better interpret this interaction. Bivariate correlations were used to verify the strength and direction of the relationship between memory performance and peritraumatic distress according to genotype. Significant negative correlations were found for individuals with the dominant homozygous genotypes: *AA* at rs3800373 *(r* = −0.69, *p* = 0.02), *CC* at rs9470080 (*r* = −0.69, *p* = 0.03), *CC* at rs1360780 (*r* = −0.69, *p* = 0.02) and *GG* at rs9296158 (*r* = −0.69, *p* = 0.02). No significant correlations were found for the other genotypes at rs3800373 (*r* = 0.39, *p* = 0.35), rs9470080 (*r* = 0.30, *p* = 0.40), rs1360780 (*r* = 0.35, *p* = 0.35), and rs9296158 (*r* = 0.35, *p* = 0.35).

Significant partial correlations were also found between the new “G × PDEQ” (*i.e*., peritraumatic dissociation) variable and memory performance for the 4 SNPs: rs3800373 *(r* = 0.51, *p* = 0.04), rs9470080 (*r* = 0.52, *p* = 0.03), rs1360780 (*r* = 0.50, *p* = 0.04), and rs9296158 (*r* = 0.50, *p* = 0.04). Bivariate correlations were again used to verify the strength and direction of the relationship between memory performance and peritraumatic dissociation according to genotype. Significant negative correlations were also found between the new “G × PDEQ” variable and memory performance for individuals with the dominant homozygous genotypes: *AA* at rs3800373 (*r* = −0.67, *p* = 0.03), *CC* at rs9470080 (*r* = −0.68, *p* = 0.03), *CC* at rs1360780 (*r* = −0.67, *p* = 0.03) and *GG* at rs9296158 (*r* = −0.67, *p* = 0.03). No significant correlations were found for the other genotypes at rs3800373 (*r* = 0.14, *p* = 0.75), rs9470080 (*r* = 0.04, *p* = 0.91), rs1360780 (*r* = 0.03, *p* = 0.94), and rs9296158 (*r* = 0.03, *p* = 0.94).

These results support an interactive effect of FKBP5 SNPs and subjective trauma severity on memory performance only for individuals homozygous for the dominant allele of the FKBP5 SNPs. In other words, people homozygous for the dominant allele and experiencing higher levels of peritraumatic responses showed poorer memory performance.

## 4. Discussion

In line with our first and second hypotheses, the FKBP5 gene is (i) associated with PTSD diagnostic status measured 9 months after trauma-exposure, and (ii) prospectively predicts lack of improvement among treatment-seeking individuals with acute PTSD. This is an important finding in as much as PTSD is increasingly considered as a disorder of failed recovery [3]. The assocation between FKBP5 and unremitting PTSD is consistent with previously published studies [8,12,13,15] and extends to specify that the gene is associated with the persistence of the disorder. Our pattern of findings is however inconsistent with the previous studies where the *C* allele of rs3800373, the *T* allele of rs9470080, the *T* allele of rs1360780 and the *A* allele of rs9296158 were associated with higher PTSD symptoms. In the present study, the *AA* gentotype of rs3800373, the *CC* genotype of rs9470080, the *CC* genotype of rs1360780, and the *GG* genotype of rs9296158 were most often found among individuals with chronic unremitting PTSD. It should be recalled that the previously published studies recruited cross-sectional samples of trauma exposed individuals not assessed for PTSD [12], not all meeting the PTSD diagnostic criteria [13], or mixing acute, chronic, past and current PTSD [15]. In this latter study [15] race and comorbidity differed substantially from our sample. It is possible that early life trauma such as childhood abuse could also alter biological developmental processes, such as HPA-axis development, as well as interaction with genetic factors in a different way compared to traumatic events experienced in adulthood. The fact that our finding is at odds with such previous results is, therefore, not very surprising. If taken together, the studies published on the FKBP5 gene suggest a role for these polymorphisms, the allele(s) responsible for this awaits further replication in other samples using a longitudinal methodology that differentiates between the development and persistence of PTSD.

In line with our third hypothesis, analyses also revealed an interactive effect of FKBP5 polymorphisms and subjective trauma severity (*i.e*., peritraumatic responses) on memory performance only for participants carrying the *AA* genotype of rs3800373, the *CC* genotype of rs9470080, the *CC* genotype of rs1360780, and the *GG* genotype of rs9296158, but not for participants with other genotypes. In other words, individuals homozygous for the dominant allele, which were most often people suffering from chronic unremitting PTSD, and having experienced higher levels of peritraumatic distress and dissociation, had poorer memory performance. This finding is consistent with previous results suggesting a relationship between corticosteroid concentration and memory performance [10], which could interfere with extinction learning, and therefore explain, in part, the persistence of the disorder. 

Results from the present study enhance our understanding of the genetics of PTSD. It provides information about factors contributing to the persistence of the disorder. These findings were obtained despite a small sample size, in part because we used a more powerful longitudinal design that differentiated between acute and chronic PTSD, which other studies had failed to do so far. According to these preliminary results as well as previous results, the FKBP5 could be associated with the development and the persistance of PTSD. It will also be up to future studies to clarify whether/how the SNPs relate to corticosteroid receptor function and cortisol signaling.

### 4.1. Study Limitations

It could be argued that the group who remitted simply had better psychologists or received a better treatment (a factor which we did not control for), but if that was the case, findings of group differences at the genetic level would not be expected.

Because of the small sample size, some correlations may be less stable. No correction was used for multiple testing, thereby inflating the risk of committing a Type I error. It would be important to replicate this work in a larger sample and include a group of trauma-exposed individuals who did not develop the disorder. Although our findings are preliminary, we believe they are of special interest for researchers interested in understanding the neurobiological mechanisms that influence psychological responses to extreme stress and their treatment. Perhaps in the future, it will be possible to predict response to treatment among sufferers of PTSD and tailor such treatments according to their genetic makeup.
